# Gastric Cancers Missed at Upper Endoscopy in Central Norway 2007 to 2016—A Population-Based Study

**DOI:** 10.3390/cancers13225628

**Published:** 2021-11-10

**Authors:** Marianne Beck, Erling A. Bringeland, Gunnar Qvigstad, Reidar Fossmark

**Affiliations:** 1 Department of Clinical and Molecular Medicine, Faculty of Medicine, Norwegian University of Science and Technology (NTNU), 7491 Trondheim, Norway; maribeck@stud.ntnu.no (M.B.); erling.audun.bringeland@stolav.no (E.A.B.); gunnar.qvigstad@stolav.no (G.Q.); 2 Department of Gastrointestinal Surgery, St Olav’s Hospital, Trondheim University Hospital, Prinsesse Kristinas Gate 1, 7030 Trondheim, Norway; 3 Department of Gastroenterology, St Olav’s Hospital, Trondheim University Hospital, Prinsesse Kristinas Gate 1, 7030 Trondheim, Norway

**Keywords:** gastric cancer, missed cancer, upper endoscopy

## Abstract

**Simple Summary:**

Stomach cancer may be missed during upper endoscopy. We have examined how often this occurs and identified factors associated with missed cancers. Among 730 patients with gastric cancer, 67 (9.2%) were missed during endoscopy 6 to 36 months prior to the cancer diagnosis. Missed cancers were more often located in the upper part of the stomach, of Lauren’s diffuse histologic type and more frequent in patients with previous Billroth II operation. The missed cancers were diagnosed at somewhat earlier stages than the non-missed cancers. In missed cancers, an ulceration was more often found in patients with shorter time interval between the first endoscopy and the endoscopy where the cancer was diagnosed. The factors associated with missed stomach cancers should be kept in mind by doctors performing endoscopies as this may lead to an earlier diagnosis of cancer.

**Abstract:**

Background: The rates of missed gastric cancers (MGC) at upper endoscopy (UE) has been reported at 5–10% in Western countries. We aimed to calculate the rate of MGC and identify factors associated with MGC. Methods: Retrospective population-based cohort study including 730 patients diagnosed with gastric adenocarcinoma in Central Norway 2007–2016. MGCs were incident gastric adenocarcinomas diagnosed 6–36 months after a previous UE. Factors associated with MGC were examined. Definitely missed (UE 6–12 months prior) and potentially missed (UE 12–36 months prior) MGCs were compared. Results: Sixty-seven (9.2%) of 730 gastric cancers were MGC. MGC were associated with localization (*p* = 0.009) and more frequent in the corpus, Lauren’s histological type (*p* = 0.028) and diffuse type more prevalent, and previous Billroth 2-operation (14.9% vs. 4.7%, *p* = 0.001). MGCs were diagnosed at earlier stages (*p* = 0.037). An ulceration was more common in patients with definitely missed than potentially MGC (40.9% vs. 17.8%, *p* = 0.041). Conclusions: MGC accounted for 9.2% of gastric cancers in Central Norway. MGC were associated with localization in the corpus, Lauren´s diffuse type and previous Billroth-2-operation. Intensified follow-up and adequate biopsy sampling of patients with gastric ulcerations could reduce the rate of missed gastric cancers.

## 1. Introduction

Gastric adenocarcinoma is one of the most common cancers worldwide [1]. In Western populations, there has been a steep decline in incidence of adenocarcinomas of Lauren’s intestinal type, paralleling the prevalence of *Helicobacter pylori* (*H. pylori*) [2,3]. However, the incidence of Lauren’s diffuse-type cancers has increased over several decades [2], and there is also an increasing incidence of non-cardia gastric cancers in younger cohorts, particularly those located in the gastric corpus and fundus [4]. Gastric cancer has a high lethality, and early diagnosis is the most important factor that affects survival. However, the majority of the patients are diagnosed with advanced disease, and surgery is curative in only a minor proportion [5]. Screening programs have therefore been implemented in countries with a relatively high incidence of gastric cancer, where individuals at moderate to high risk are examined with upper endoscopy to diagnose early gastric cancer and preneoplastic lesions [6]. The use of high-definition endoscopes, enhanced imaging techniques [7,8] and longer inspection time [9] are associated with higher detection rates of both cancer and preneoplastic lesions [6,7,8]. A clinically relevant and highly robust quality indicator of upper endoscopies is the diagnosis of cancer after a procedure. The term interval gastric cancer encompasses cancers diagnosed between scheduled endoscopies within a screening program, whereas in Western countries without a screening program, cancers diagnosed within a defined period after upper endoscopy have been termed missed gastric cancer (MGC) [10,11]. The rates of MGC have previously been reported between 4.7% and 9.8% in Western countries [10,12]. It is therefore of interest to identify factors associated with MGC, and characteristics of the gastric lesion, the patient and the endoscopist have been proposed [13]. The aim of this population-based study was to calculate the rate of missed gastric cancers in Central Norway as well as to identify factors associated with missed gastric cancers.

## 2. Materials and Methods

### 2.1. Study Design and Data Source

A retrospective cohort study was performed using a database described in previous publications [5,14,15]. The patient cohort consisted of 730 consecutive patients diagnosed with gastric cancer in Central Norway between January 2007 and December 2016. The catchment area of 700,000 persons comprised some 14% of the Norwegian population. Patients were identified through an initial search in the Norwegian Cancer Registry (NCR) and Norwegian Patient Registry (NPR) databases using ICD-10 codes C16.0 to C16.9 and C15.5/C15.9. The results from the two searches were merged based on a unique 11-digit identification number for each citizen in Norway. Patients with tumors other than gastric adenocarcinoma were excluded by manual assessment of all patient records. Patients with Siewert type I cancer were excluded, whereas patients with type II and III cancers were retained for further analyses. Clinical and histopathological characteristics were recorded as previously described [5,14,15]. A dedicated pathologist reviewed histological sections from all patients to ensure a uniform histological classification according to Lauren [3]. Upper endoscopies in Central Norway from 2004 to 2016 were performed using Olympus endoscopes GIF-160/H180/Q180/HQ190. The censoring date was 20 May 2021, allowing a minimum follow-up of 4 years and 4 months.

### 2.2. Definitions

MGC were defined as incident gastric adenocarcinomas diagnosed between 6 and 36 months after a previous upper endoscopy [10,16]. The date of cancer diagnosis was defined as the date of the upper endoscopy where a lesion was described as suspicious of cancer and biopsied. Patients undergoing multiple (3 or more) upper endoscopies with biopsies in the past 6 months before the final diagnosis of gastric cancers were not perceived as missed cancers. The patient group without missed cancers was defined as a control group. The MGCs were further subdivided into “definitely missed” and “potentially missed” cancers, defined as upper endoscopy being performed 6–12 months or 12–36 months prior to date of diagnosis, respectively [12,17].

### 2.3. Variables

The primary aim was to calculate the overall rate of missed gastric cancer. Additional analyses were performed to identify any clinical factors associated with MGCs, such as tumor localization, previous Billroth 2 surgery, Lauren’s histological subtype [3] and TNM stage [18]. Tumor location was categorized as cardia (type II and III), corpus (including fundus), antrum or diffuse. The time interval between previous upper endoscopy and time of diagnosis was calculated. Findings at the endoscopy prior to the date of diagnosis were recorded, as well as indication for endoscopy and the presence of alarm symptoms (dysphagia, gastrointestinal bleeding, anemia, vomiting and general/constitutional symptoms).

### 2.4. Data Collection

Medical records of the patient cohort with gastric cancer were searched electronically and manually to identify upper endoscopies performed 6–36 months before the diagnosis of cancer. The following NOMESCO Classification of Surgical Procedures (NCSP), procedure codes were used for electronic and manual searches: JUD02, JUD05, UJD02 and UJD05 [19]. In addition, the medical records of the past 36 months prior to the cancer diagnosis were reviewed manually to search for endoscopy reports with missing procedure codes.

### 2.5. Statistical Analysis

Continuous variables are presented as median (range) or mean ± standard deviation (SD) depending on distribution and analyzed using the Mann–Whitney or Student’s t test. Categorical variables were cross tabulated and analyzed by χ² or Fischer exact test to identify differences between groups. *p* values < 0.05 were considered significant. Overall survival was calculated using the Kaplan–Meier method. The Cox proportional hazard method was used in a multivariable analysis. Statistical analyses were conducted using SPSS version 27 (IBM, Armonk, NY, USA).

### 2.6. Ethics Approval

The gastric cancer projects have been approved by the Regional Committee for Medical and Health Research Ethics of Central Norway (2011/1436) in accordance with the principles of the Declaration of Helsinki.

## 3. Results

**Figure 1:**
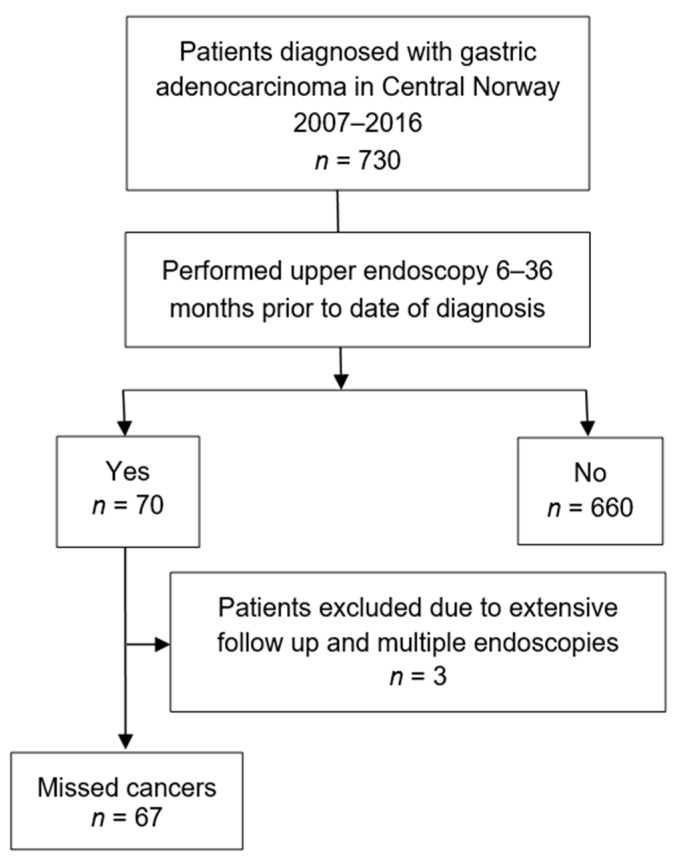
Flow diagram of study population.

Among the 730 patients constituting the study cohort, 70 (9.6%) had undergone an upper endoscopy 6 to 36 months prior to the date of the cancer diagnosis. Three patients underwent intensive follow-up with multiple endoscopies within the last six months before cancer diagnosis and these were not perceived as missed cancers and hence included in the control group consisting of 663 patients. A total of 67 patients (9.2%) were assigned to the missed cancer group and were the objective for further analyses (Figure 1).

### 3.1. Patient and Characteristics

Median age of the entire cohort was 73.8 (21.1–98.5) years, with 461 (63.2%) males. Age and sex did not differ significantly between missed cancers and controls (Table 1).

### 3.2. Tumour Localization and Stage

The tumor localization differed significantly between MGC and controls (*p* = 0.009), which was explained by a higher proportion of MGC localized in the gastric corpus (44.8% vs. 25.3%) (Table 1). None of the patients with MGC had previously been operated with gastric bypass surgery or gastric sleeve resection. Overall, neither the T stage nor the TNM stage distribution differed between missed cancers and controls (*p* = 0.22), but taking advantage of stage as an ordinal variable, a linear-by-linear test returned a p value of 0.031, indicating a trend for the missed cancers to be diagnosed at earlier stages. This is further supported by an χ² test on the merged groups (Stage 0–3) vs. (Stage 4 + X), with a lower proportion of the latter in the missed cancer group, 43% vs. 56.6% (*p* = 0.037). Stage 4 is incurable metastatic disease, whereas stage X for practical purposes indicates lack of staging due to advanced age and comorbidity, both entities with inferior long-term survival rates.

### 3.3. Lauren Classification

The Lauren classification differed between missed cancers and controls (*p* = 0.028) (Table 1), with MGC more often being of diffuse type compared to controls (37.3% vs. 28.5%) and less frequently of intestinal type (31.3% vs. 43.7%).

### 3.4. Previous Billroth-2-Anastomosis

Of the patients in the missed cancer group, 10 (14.9%) were previously Billroth-2-resected, versus 31 (4.7%) in the control group (*p* = 0.001).

### 3.5. Definitely Missed versus Probably Missed Cancers

Thirty-seven (55.2%) of the 67 missed cancers had alarm symptoms at the endoscopy prior to the endoscopy leading to diagnosis (Table 2). The mean time from upper endoscopy to date of diagnosis was 17.5 (±8.8) months. Among the missed cancers 22 (32.8%) of 67 were definitely missed cancers whereas the remaining 45 (67.2%) were potentially missed cancers. In the definitely missed group 9/22 (40.9%) had an ulceration at the prior upper endoscopy, while 8/45 (17.8%) in the potentially missed group had an ulceration (*p* = 0.041). There were no statistically significant differences for other findings at upper endoscopy, cancer localization or Lauren distribution.

### 3.6. Indications for Upper Endoscopies Prior to Diagnosis versus at Diagnosis

The symptoms or indication for the endoscopies prior to the diagnosis were compared to the symptoms or indication for the endoscopy at diagnosis (Table 3). Only weight loss differed significantly between the two time points; four (6.0%) patients versus 18 (26.9%) patients prior and at diagnosis, respectively, had this symptom.

### 3.7. Biopsy Sampling of Ulcerations

Seventeen patients (25.4%) in the MGC group had an ulceration at the prior upper endoscopy. In 9 (53.0%) of these patients no biopsy was taken from the ulceration. In 3 (17.6%) patients the biopsy was taken but pathology reports were missing from the patients’ medical records and could not be retrieved. Three (17.6%) biopsies were described as normal/unspecified inflammation and two (11.8%) biopsies contained mild to moderate dysplasia.

### 3.8. Endoscopist Experience

Endoscopist experience at the endoscopy prior to diagnosis and at the endoscopy leading to diagnosis were compared. At the endoscopies prior to the diagnosis, 21 (31.3%) were performed by junior doctors and 46 (68.7%) by senior doctors. The endoscopy leading to the cancer diagnosis was performed by junior doctors in 15 (22.4%) of the patients, whereas 52 (77.6%) were examined by a senior doctor. This difference was not statistically significant (*p* = 0.330).

### 3.9. Survival

**Figure 2:**
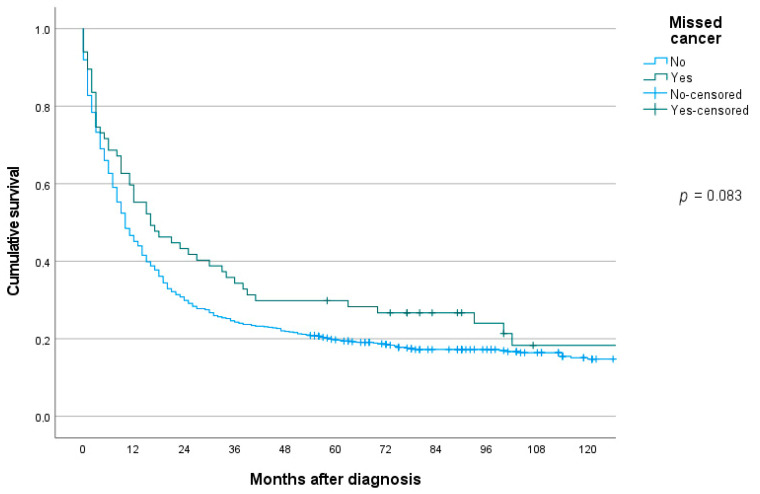
Long-term overall survival in patients with gastric cancer missed at upper endoscopy or not.

The median survival in patients with missed gastric cancer was significantly better compared to controls, 16 months (95% CI 5.7–26.3) vs. 10 months (95% CI 8.3–11.7), *p* = 0.036. Kaplan–Meier survival curves showed a similar, but non-significant trend, log-rank test *p* = 0.083 (Figure 2). In a Cox multivariable analysis adjusting for age, sex, TNM stage, Lauren type and localization, interval cancer was not significantly associated with risk of death, HR 1.16 (95% CI 0.87–1.55), *p* = 0.327.

## 4. Discussion

In this population-based study, we found an MGC rate of 9.2%. Cancer localization in the gastric corpus, Lauren diffuse-type histology and previous Billroth-2-operations were more prevalent among patients with MGC. At the upper endoscopy prior to MGC diagnosis, an ulceration was found in a higher proportion of patients with definitely missed compared to potentially missed gastric cancer. MGCs were diagnosed at an earlier stage and patients with MGCs tended to have improved survival compared to the control group. The strengths of the present study include its population-based design comprising all patients diagnosed with gastric adenocarcinoma in Central Norway during a 10-year period. Furthermore, the study had a near complete follow-up. However, the study was limited by its retrospective design. The large majority of patients at the age where gastric cancer is diagnosed, are traditionally examined at public hospitals, but some endoscopic workup by private health care providers for the relevant patient group cannot, per principle, be ruled out, and the percentage of missed cancers could be slightly higher than that reported.

### 4.1. MGC Rate

The rate of MGC in Central Norway of 9.2% was comparable to three Western studies that reported MGC rates of 4.7% to 9.8% after 36 months [10,12,16]. A meta-analysis of 22 studies with heterogenous design found that 9.4% of gastric cancers were missed at upper endoscopy [13]. MGC in these studies was defined as gastric cancer diagnosed from 3–12 to 36–42 months after a previous upper endoscopy [11,16,20] a variation that would clearly affect the reported MGC rates. The use of three years in the definition of MGC derives from a Japanese landmark study from the 1970s that suggested a doubling time for mucosal gastric carcinoma of 2–3 years [21], whereas it is less than one year for more advanced cancers [22]. In general, Eastern studies have reported higher rates of missed or interval gastric cancer than Western studies, which may reflect differences in histologic criteria as early cancers in Japan often are categorized as dysplastic lesions by Western pathologists [11]. The proportion of gastric cancers that are MGCs in populations without a screening program will, in addition to the quality of the endoscopy, also be influenced by how widespread upper endoscopies are used to investigate symptoms from the upper gastrointestinal tract.

### 4.2. Risk Factors of MGC

Several risk factors for MGC were identified in our population. MGC were more likely to be localized in the gastric corpus compared with controls. This has been a consistent finding in several previous studies and potential reasons such as gastric folds hiding neoplasms, and that sublocations in the gastric body are difficult to visualize, have been proposed [13]. We also found that the proportion of Lauren diffuse type histology was higher in the missed cancer group compared to controls (37.3% vs. 28.5%), which has also been reported in a recent Spanish cohort [10]. These observations are highly relevant, as the incidence of the Lauren diffuse type cancers as well as cancers in younger age cohorts located to the corpus are increasing [2,4]. The association between histological differentiation and missed cancers is inconsistent [10,23], but poorly differentiated cancers were more frequent among MGCs in a meta-analysis [13]. We did not find that sex or age differed between patients with MGC and controls, whereas a study from the United Kingdom reported MGC to be more common in younger patients and females [11]. The same study found that early cancers were more frequent in the missed cancer group. We observed that a higher proportion of MGC were diagnosed in less advanced TNM-stages, which could be merely a consequence of that many lesions overlooked at the endoscopy prior to the cancer diagnosis were small. Patients with a previous Billroth-2-operation were more prevalent in the MGC than in controls. This finding has been reported in one previous publication [10], suggesting that stump cancers may be difficult to detect by visual inspection during endoscopy. However, Billroth-2-resected patients may have been part of a follow-up program and may have undergone regular upper endoscopies, which could also contribute to a higher frequency of cancers defined as MGC in this group. It is also known that Billroth-2 resected patients may have H pylori infection and are exposed to duodenogastric reflux that increase the risk of gastric cancer [24]. We found a higher ulcer frequency in the definitely missed cancer group compared to the potentially missed cancer group. Similarly, others have reported that marked gastric atrophy and gastric ulcer were predictive factors for missed gastric cancers [10,11,13] and inconclusive histology reports may also be a contributor [11]. Factors concerning the quality of the endoscopy have been implicated, such as inability to detect lesions, taking an insufficient number of biopsies from detected lesions, inappropriate follow-up of detected lesions and various technical limitations [13]. A Scottish cohort study found that 9.8% were missed cancers and concluded that errors by the endoscopist accounted for the majority of cases [12]. In a United States population, MGC were associated with the endoscopy being performed by a non-gastroenterologist and the procedure performed in outpatients [16].

Alarm symptoms at the prior upper endoscopy were reported in 50.0% of our patients in the definitely missed cancer subgroup, which did not differ significantly from the subgroup with possibly missed cancers. An Australian study from 2010 found a similar rate of alarm symptoms at 59% among patients with MGC [17], whereas others have found that lack of alarm symptoms was more frequent in patients with MGC [10]. When comparing the symptoms at the prior upper endoscopy with symptoms at diagnosis, only weight-loss was significantly more common at diagnosis, which is a well-known alarm symptom. However, a proportion of small cancers has not caused symptoms per se and has been incidental findings. This is supported by the fact that some cancers were diagnosed due to scheduled follow-up of asymptomatic conditions.

### 4.3. Survival

The consequences of overlooking gastric cancers at endoscopy can be studied in countries where images have been systematically taken during endoscopies. It seems that most cancers that were overlooked in a Japanese population were still resectable early gastric cancers two years later [25]. However, these findings cannot be extrapolated to populations where the missed cancers may be more advanced, and it is of interest to examine the survival in patients with missed gastric cancers. None of the European studies [10,11,20] have found that MGC have a worse prognosis compared to control groups. In the present study, there was a trend toward better long-term survival rates in the missed cancer group—median survival 16 months vs. 10 months in the control group, *p* = 0.036, and log-rank test for the univariable Kaplan–Meier curves, *p* = 0.083. In a multivariable Cox regression analysis, type of group (MGC vs. controls) was not related to long-term survival rates, with HR for death 1.16 (95% CI 0.87–1.55), *p* = 0.327. These observations may suggest that MGC do not have a particular aggressive biology per se, and that any tendency of better survival rates compared to the control group may be contingent on its mode of presenting, that is, a tendency toward detection at earlier stages and less frequently stage IV or stage X disease at time of diagnosis in the MGC group.

## 5. Conclusions

A total of 9.2% of the patients diagnosed with gastric adenocarcinoma in Central Norway 2007–2016 were MGC. MGC were associated with localization in the gastric corpus, histological Lauren diffuse-type and previous Billroth 2-operation. Of the patients having an upper endoscopy within the 6–12 months prior to the date of diagnosis, 40% had an ulceration. More thorough inspection of the gastric corpus during endoscopy and intensified follow-up with biopsy sampling of patients with ulcers could reduce the rate of missed gastric cancers. Long-term survival rates for missed gastric cancer patients was on par with that of the non-missed cancer group.

## Figures and Tables

**Table 1:** Patient characteristics.

Variable	Entire Cohort	Missed Cancers	Control Group	*p* Value
Patients, *n* (%)	730 (100.0)	67 (9.2)	663 (90.8)	
Age at diagnosis, years				0.619
Median (range)	73.8 (21.1–98.5)	73.0 (46.0–94.5)	73.8 (21.1–98.5)	
Sex, *n* (%)				0.728
Male	461 (63.2)	41 (61.2)	420 (63.3)	
Cancer localization, *n* (%)				0.009
Cardia	225 (30.8)	15 (22.4)	210 (31.7)	
Corpus	198 (27.1)	30 (44.8)	168 (25.3)	
Antrum	220 (30.1)	16 (23.9)	204 (30.8)	
Diffuse	84 (11.5)	6 (9.0)	78 (11.8)	
T stage, *n* (%)				0.620
T0 + Tis	29 (4.0)	3 (4.5)	26 (3.9)	
T1	68 (9.3)	10 (14.9)	58 (8.7)	
T2	35 (4.8)	3 (4.5)	32 (4.8)	
T3	114 (15.6)	10 (14.9)	104 (15.7)	
T4a	103 (14.1)	12 (17.9)	91 (13.7)	
T4b	33 (4.5)	2 (3.0)	31 (4.7)	
Tx	344 (47.1)	27 (40.3)	317 (47.8)	
TNM stage, *n* (%)				0.224
Stage 0 + 1	108 (14.8)	15 (22.4)	93 (14.0)	
Stage 2	107 (14.7)	12 (17.9)	95 (14.3)	
Stage 3	111 (15.3)	11 (16.4)	100 (15.1)	
Stage 4	310 (42.5)	21 (31.3)	289 (43.6)	
Stage X	94 (12.9)	8 (11.9)	86 (13.0)	
Lauren classification, *n* (%)				0.028
Diffuse	214 (29.3)	25 (37.3)	189 (28.5)	
Intestinal	311 (42.6)	21 (31.3)	290 (43.7)	
Mixed diffuse/intestinal	78 (10.7)	5 (7.5)	73 (11.0)	
Cancer NUD	101 (13.8)	16 (23.9)	85 (12.8)	
No malignant biopsy	14 (1.9)	-	14 (2.1)	
No biopsy	12 (1.6)	-	12 (1.8)	
Previous B2-operation, *n* (%)	41 (5.6)	10 (14.9)	31 (4.7)	0.001

UE: upper endoscopy; NUD: non-numerical unstructured data; B2: Billroth 2 operation.

**Table 2:** Characteristics of gastric cancers diagnosed 6–12 months versus 12–36 months after a previous upper endoscopy.

Variable	Missed Cancers (6–36 mo.)	Definitely Missed (6–12 mo.)	Potentially Missed (12–36 mo.)	*p* Value
Patients, *n* (%)	67 (100.0)	22 (32.8)	45 (67.2)	
Time from UE to diagnosis in months, mean (SD)	17.5 (8.8)	7.9 (2.0)	22.2 (6.8)	0.000
Alarm symptoms, *n* (%)	37 (55.2)	11 (50.0)	26 (57.8)	0.584
Findings at UE *				
Normal, *n* (%)	18 (26.9)	5 (22.7)	13 (28.9)	0.593
Gastritis, *n* (%)	26 (38.8)	8 (36.4)	18 (40.0)	0.774
Ulceration, *n* (%)	17 (25.4)	9 (40.9)	8 (17.8)	0.041
GERD, *n* (%)	14 (20.9)	6 (27.3)	8 (17.8)	0.369
Other, *n* (%)	16 (23.9)	5 (22.7)	11 (24.4)	0.877
Cancer localization, *n* (%)				0.850
Cardia	15 (22.4)	6 (27.3)	9 (20.0)	
Corpus	30 (44.8)	10 (45.5)	20 (44.4)	
Antrum	16 (23.9)	4 (18.2)	12 (26.7)	
Diffuse	6 (9.0)	2 (9.1)	4 (8.9)	
Lauren classification, *n* (%)				0.499
Diffuse	25 (37.3)	6 (27.3)	19 (42.2)	
Intestinal	21 (31.3)	9 (40.9)	12 (26.7)	
Mixed diffuse/intestinal	5 (7.5)	1 (4.5)	4 (8.9)	
Cancer NUD	16 (23.9)	6 (27.3)	10 (22.2)	

UE: upper endoscopy; SD: standard deviation; GERD: gastroesophageal reflux disease; NUD: non-numerical unstructured data. * Some patients had more than one finding.

**Table 3:** Symptoms or indication for upper endoscopy prior to the diagnosis of gastric cancer versus at the time of diagnosis in 67 patients with missed gastric cancer at the first endoscopy.

Symptom/Indication	Endoscopy Prior to Diagnosis (*n* = 67)	Endoscopy at Diagnosis (*n* = 67)	*p* Value *
GI-bleeding, *n* (%)	25 (37.1)	24 (35.8)	0.858
Upper abdominal pain, *n* (%)	15 (22.4)	18 (26.9)	0.545
Nausea/vomiting, *n* (%)	7 (10.5)	8 (11.9)	0.784
Ulcer follow-up, *n* (%)	6 (9.0)	6 (9.0)	0.762
Barrett’s follow-up, *n* (%)	5 (7.5)	1 (1.5)	0.208
B2 follow-up, *n* (%)	5 (7.5)	2 (3.0)	0.441
GERD/dyspepsia, *n* (%)	4 (6.0)	12 (17.9)	0.059
Weight loss, *n* (%)	4 (6.0)	18 (26.9)	0.002
Search for primary cancer, *n* (%)	1 (1.5)	3 (4.5)	0.619
Gastritis follow-up, *n* (%)	1 (1.5)	1 (1.5)	1
Search for primary cancer, *n* (%)	1 (1.5)	3 (4.5)	0.619
Gastric NET follow-up, *n* (%)	1 (1.5)	1 (1.5)	1
Preoperative before gastric bypass, *n* (%)	1 (1.5)	0	1
Jaundice, *n* (%)	0	1 (1.5)	1
Polyp follow-up, *n* (%)	0	2 (3.0)	0.496
Diarrhea, *n* (%)	0	1 (1.5)	1

* not adjusted for multiple testing. GI: gastrointestinal; B2: Billroth 2; GERD: gastroesophageal reflux disease; NET: neuroendocrine tumor.

## Data Availability

The original data cannot be made publicly available.
